# *Herpesvirus* Infections of the Corneal Endothelium

**DOI:** 10.3390/microorganisms13040778

**Published:** 2025-03-28

**Authors:** Jessie Wang, Behnam Rabiee, Chandani Patel, Mansab Jafri, Hamad Hussain, Aaila Chaudhry, Imtiaz Chaudhry, Layla Kamoun, Iftikhar Chaudhry, Lewis Oh, Fatima I. Bobat, Deepak Shukla, Asim V. Farooq

**Affiliations:** 1 Duke Eye Center, Duke University, Durham, NC 27705, USA; jessiewang13@gmail.com; 2 Department of Ophthalmology, Nazareth Hospital, Trinity Health Mid-Atlantic, Philadelphia, PA 19152, USA; behnam.rabiee@gmail.com (B.R.); cpatel@willseye.org (C.P.); aailaijaz@yahoo.com (A.C.); ifmc2004@yahoo.com (I.C.); kamoun.usa@gmail.com (L.K.); ifmc@comcast.net (I.C.); 3 IC Laser Eye Care, Bensalem, PA 19020, USA; mansab.jafri@gmail.com (M.J.); hamadhussainmd@gmail.com (H.H.); 4 Rowan-Virtua School of Osteopathic Medicine, Rowan University, Stratford, NJ 08084, USA; 5 Pritzker School of Medicine, The University of Chicago, Chicago, IL 60637, USA; sanghyon@uchicagomedicine.org; 6 Department of Ophthalmology and Visual Sciences, The University of Illinois Chicago, Chicago, IL 60607, USA; fbobat2@uic.edu (F.I.B.); dshukla@uic.edu (D.S.); 7 Department of Ophthalmology and Visual Science, The University of Chicago, Chicago, IL 60637, USA

**Keywords:** corneal endotheliitis, corneal edema, herpetic endotheliitis, herpesviridae, HSV endotheliitis, VZV endotheliitis, CMV endotheliitis, EBV endotheliitis

## Abstract

Corneal endotheliitis is an inflammatory process, most commonly of viral etiology, that manifests clinically with features including corneal edema, keratic precipitates, and a mild anterior chamber reaction. Several studies have implicated human herpesviruses from the *Herpesviridae* family as primary causes of corneal endotheliitis, including cytomegalovirus (CMV), varicella zoster virus (VZV), and herpes simplex viruses 1 and 2 (HSV-1 and HSV-2). This review critically evaluates the present literature surrounding herpesvirus infections of the corneal endothelium.

## 1. Introduction

Corneal endotheliitis is broadly defined as inflammation of corneal endothelial cells (CECs), typically presenting with keratic precipitates (KPs) and corneal edema with a mild anterior chamber reaction [1]. Numerous reports have suggested a viral etiology for corneal endotheliitis [1,2,3,4,5,6,7]. These include human herpesviruses from the *Herpesviridae* family, including cytomegalovirus (CMV), varicella zoster virus (VZV), and herpes simplex viruses 1 and 2 (HSV-1 and HSV-2) [1,2,3,4,5,6,7]. In particular, *Herpesviridae* corneal endotheliitis (HCE) may be suspected among those with a history of prior ocular infection, although clinical examination findings and treatment response (or a lack thereof) can be suggestive.

Considering that CECs have a limited ability to regenerate [8], prompt diagnosis is essential to prevent permanent endothelial cell loss and corneal compromise [2]. Limitations in non-invasive diagnostic techniques likely contribute to an underdiagnosis of this entity. Viral serologies may be of limited utility given the wide prevalence of these viruses among humans, although negative testing can be helpful to rule out an infectious etiology [1]. Improving our understanding of the pathophysiology of HCE, including the mechanism(s) of viral entry, is critical to improving diagnostic and therapeutic strategies. Herein, we aim to provide an overview of HCE, including pathophysiology, clinical manifestations, diagnostic techniques, and management, and also provide suggestions for future directions.

## 2. Methods

A review of the literature was conducted using the PubMed database. Manuscripts in English or with English translation were included, and articles unavailable in English, considered off-topic, or with unclear methodology were excluded. The PubMed search query included “herpetic endotheliitis”, “HSV endotheliitis”, “VZV endotheliitis”, “CMV endotheliitis”, and “EBV endotheliitis”. Further references were included through the exploration of the cited sources of the initial search results.

## 3. Results

### 3.1. Cytomegalovirus (CMV)

#### 3.1.1. Epidemiology and Pathophysiology of CMV Endotheliitis

CMV is a double-stranded DNA virus transmitted through direct contact with infected bodily fluids. Seroprevalence rates vary from 45 to 100% across the world, with higher rates found in Asia, South America, and Africa, and lower rates recorded in Europe and the United States. Independent from geographic differences, its prevalence has also been found to be inversely related to socioeconomic status [9]. CMV endotheliitis is most commonly seen in middle-aged and older men (mean age of 66.9 years, 80.2% males), with the majority of reports involving Chinese and Japanese patient populations [10,11]. Like other herpes viruses, CMV infection is typically characterized by an asymptomatic primary infection, after which the virus establishes lifelong latency in the bone marrow stem cells and myeloid cells. The virus can then periodically reactivate, causing recurrent disease [12].

**Figure 1:**
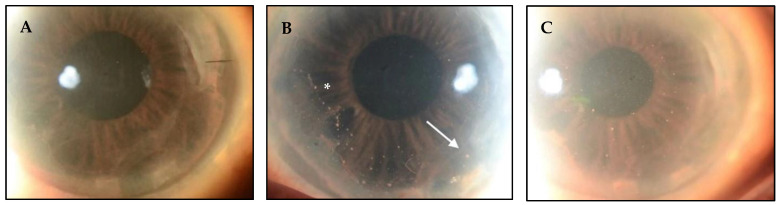
CMV endotheliitis following DMEK. (**A**) Clear DMEK graft after procedure. (**B**) Linear pattern of KPs (white asterisk) with one large KP (white arrow) can be seen on the host endothelium near the graft host junction, which differentiates this from allograft rejection. (**C**) Reduction in the number and size of KPs following anti-CMV therapy (Courtesy of Kumar et al. Used with the permission of the publisher [14]).

In immunocompetent individuals, ocular manifestations of CMV primarily occur in the anterior segment, while posterior segment involvement is more common in those who are immunocompromised. Recent studies have found that many patients diagnosed with CMV endotheliitis have previously undergone corneal transplants. This raises the conjecture that local immunosuppression secondary to topical steroids may increase the risk for CMV, and that CMV may contribute to graft failure and/or endothelial dysfunction (Figure 1) [13].

Glycoproteins remain important for viral entry and immune recognition of herpesvirus infections. The gB complex is essential for cell-to-cell spread in CMV, while the gM/gD complex mediates the attachment of the virus to host cells. Although cell entry receptors for CMV have been poorly understood, studies have implicated EGFR, CD90, and NRP2 in epithelial cells [3,4,5,6,7,8,9,10,11,12,13,14,15,16,17,18,19,20,21,22,23,24,25,26,27,28,29,30,31,32,33,34,35,36,37,38,39,40,41,42,43,44,45,46,47,48,49,50,51,52,53,54,55,56,57,58,59,60,61,62,63,64,65,66,67,68,69,70,71,72,73,74,75,76,77,78,79,80,81,82,83,84,85,86,87,88,89,90,91,92,93,94,95]. In a manner similar to that of HSV, the CMV virus can reactivate in neuronal tissue. A small amount of viral particles can then be shed into the anterior chamber via the nerve branches supplying the trabecular meshwork, iris, and ciliary body and captured by indigenous antigen-presenting cells (APCs), which in turn induce virus-specific anterior chamber associated immune deviation (ACAID) [41,77,78,79]. ACAID describes the phenomenon that grants immune privilege to the anterior chamber and suppresses CD4, Th1/2, and B cell activities, protecting eyes from inflammatory blindness [96]. However, ACAID also impairs the immune response to viral infections and may potentially contribute to the pathogenesis of endotheliitis. CMV infects multiple cell types, including fibroblasts, via fusion at the cell surface and epithelial cells via endocytosis. Wang et al. demonstrated its entry into the retinal pigmented epithelial (RPE) cells with electron microscopy, showing that virus produced in epithelial cells preferentially fuses with the plasma membrane, whereas fibroblast-derived virus mostly enters by receptor-mediated endocytosis, suggesting that the cell type in which a CMV virus is produced in may influence its subsequent spread and pathogenesis [80,81].

#### 3.1.2. Clinical Manifestations of CMV Endotheliitis

**Figure 2:**
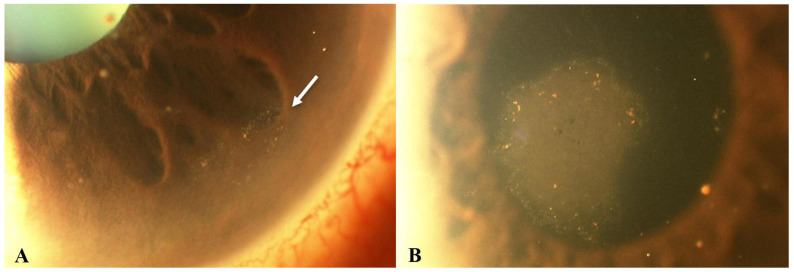
Slit lamp photos of a patient with CMV endotheliitis. (**A**) KPs in a ring configuration (white arrow) can be seen in the inferior cornea and (**B**) a coin-shaped patch of corneal endotheliitis with keratic precipitates lining the border of the lesion are visible (Courtesy of Chee et al.; used with the permission of the publisher [37]).

**Figure 3:**
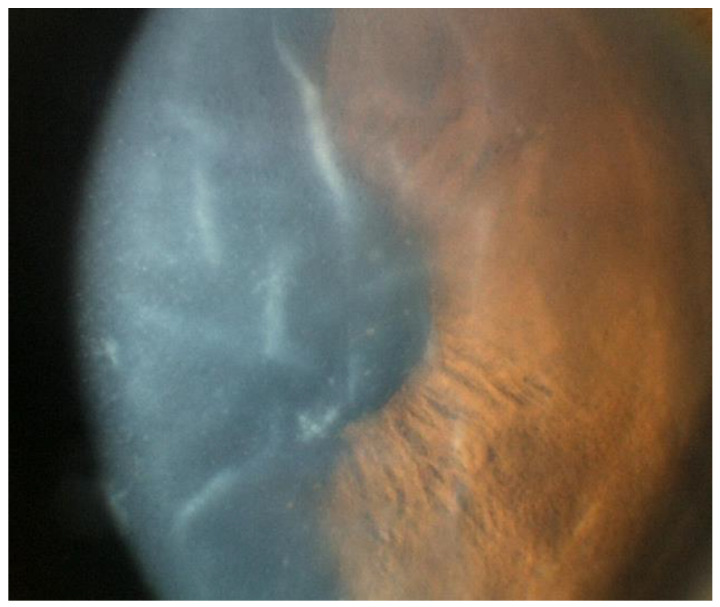
Slit lamp photo of an eye with CMV endotheliitis showing severe corneal swelling with Descemet’s folds, epithelial bullae, and medium-sized KPs (Courtesy of Chee et al.; used with the permission of the publisher [37]).

CMV may represent the most common cause of HCE. Unlike other viral endotheliitis etiologies, CMV-related endotheliitis typically arises in otherwise healthy patients with no history of compromised immune systems [1,2]. Clinical findings of CMV endotheliitis are similar to those of other viral entities, including small to medium KPs, which are often pigmented and non-granulomatous [10]. CMV-related HCE may be classified as linear, sectoral, disciform, or diffuse, depending on the distribution of the KPs and the pattern of overlying stromal and epithelial edema. Amongst the four clinical types, disciform is the most common pattern, occurring in about 70% of cases (Figure 2) [10]. In linear endotheliitis, the KPs are present in a linear distribution and corneal edema is localized (Figure 3) [10,33,34]. In sectoral endotheliitis, KPs spread across a broader region and corneal edema is also localized. Finally, in diffuse endotheliitis, KPs and corneal edema are diffuse [10,33,34].

Additional clinical symptoms that may be noted are atrophy of the iris, minimal inflammatory cells in the anterior chamber, and decreased endothelial cell count [33,34,35].

#### 3.1.3. Diagnostic Techniques of CMV Endotheliitis

As with all cases of suspected viral endotheliitis, CMV-related endotheliitis is primarily a clinical diagnosis. However, an anterior chamber tap with detection of CMV DNA via a polymerase chain reaction (PCR) in the aqueous humor can be used to confirm the diagnosis [2,36]. PCR analysis of the sample for HSV and VZV should also be performed, as these viral entities present similarly clinically. Because the aqueous humor is thought to be free of pathogens normally, a positive test result is typically a reliable indicator of infection [37]. It should be noted, however, that PCR may only result in a positive test during the early stages of infection, as a decrease in viral load to below detectable levels later in the disease course may preclude accurate detection. In a study of 53 patients with suspected viral anterior uveitis, the Bascom Palmer Eye Institute reported that performing an anterior chamber paracentesis changed management in only 7 patients [38]. Thus, the benefits of an anterior chamber tap with PCR of the aqueous humor must be weighed against the potential risk for complications from this procedure [21].

**Figure 4:**
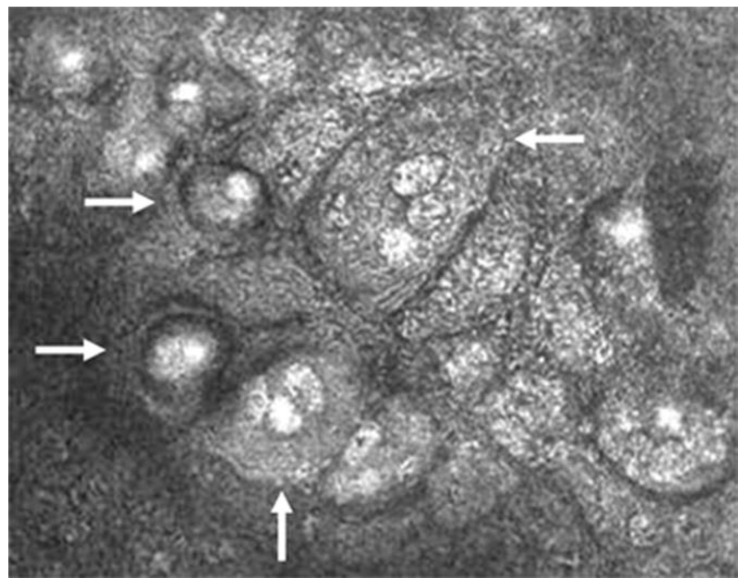
Confocal microscopic image of CMV endotheliitis at the level of corneal endothelium showing a group of large cells whose nuclei have a high reflection area surrounded by a halo of low reflection, resembling an “owl’s eye” as represented by white arrows (Courtesy of Shiraishi et al.; used with the permission of the publisher [44]).

The pathognomonic finding confirming the diagnosis of CMV is the presence of inclusion bodies and macrophages on confocal microscopy (“owl’s eye”; Figure 4) [2,33,40]. This finding is specific to CMV and is not seen in HSV, VZV, or other viral entities. Finally, anterior segment spectral-domain OCT can show high reflectivity of posterior corneal lesions, which are presumed KPs, but unlike the finding of “owl’s eyes” on confocal microscopy, this finding is not specific to CMV [41].

#### 3.1.4. Treatment of CMV Endotheliitis

As with other causes of viral endotheliitis, treatment for CMV-related endotheliitis is directed at controlling both the inflammatory and infectious components of the disease process. This is most commonly achieved by the use of a topical corticosteroid in combination with a topical and/or systemic antiviral, with studies suggesting their long-term efficacy for the preservation of endothelial function [43,45]. IOP-lowering medications may also be used in cases of ocular hypertension.

Recent reports have shown that systemic antivirals [36], specifically ganciclovir, are effective in treating CMV-related endotheliitis [43]. In 1988, ganciclovir became the first FDA-approved treatment for CMV [44], and it has since become the standard of care. Current guidelines do not report a consensus on the recommended dosages for oral treatment, but the general guideline is to administer consistent dosages at least once daily for a year [1]. If oral treatment does not suffice, intravitreal or topical therapy can be added. Systemic therapy produces a higher response rate than topical therapy but carries a greater risk for side effects; meanwhile, intravitreal ganciclovir implants also demonstrate a high efficacy rate but comes at the cost of a higher relapse rate as compared to topical ganciclovir gel. Thus, intravitreal ganciclovir may be a useful adjunct to systemic therapy while topical ganciclovir may be useful for long-term maintenance therapy [35]. Today, ganciclovir is under careful review given evidence of viral resistance in some patients [46], and in these cases of resistance, an alternative such as foscarnet may be considered [44,46].

Though dosing frequency varies in the literature, potential antiviral regimens are listed as follows: oral valganciclovir 900 mg BID with subsequent taper to prophylactic dosing at 900 mg QD (first-line systemic regimen), intravenous 5 mg/kg ganciclovir BID (second-line systemic regimen), and intravenous foscarnet 60 mg/kg TID or cidofovir 5 mg/kg (third-line systemic regimen). As foscarnet and cidofovir can cause significant nephrotoxicity, systemic medications should be ideally co-managed with an infectious disease specialist. Topical regimens include 0.15% ganciclovir gel five times daily with subsequent taper to prophylactic dosing at TID (first-line topical regimen) and compounded ganciclovir 0.5–2% (second-line topical regimen) [1,11,14,43,45,47,48,49,50].

If a patient fails medical therapy, corneal transplantation may be warranted in instances of irreversible corneal decompensation. Successful cases of endothelial keratoplasty have been reported, though there should ideally be at least a 90-day period of quiescence before proceeding with surgery to optimize surgical outcomes [14,49].

### 3.2. Varicella Zoster Virus (VZV)

#### 3.2.1. Epidemiology and Pathophysiology of VZV Endotheliitis

As a member of the *Herpesviridae* family, VZV is a double-stranded DNA virus with a 95–99% seroprevalence amongst the adult population in the United States [59,83]. Its incidence ranges from 1.2 to 3.4 per 1000 people each year in young individuals, increasing to 10–14 per 1000 people per year in those over 65 years of age. Roughly 10–20% of VZV cases involve ocular tissue due to involvement of the ophthalmic (V1) branch of the trigeminal nerve [59]. When this occurs, the disease entity is classified as herpes zoster ophthalmicus (HZO). Recent evidence suggests that HZO has increased by 23% per decade since 1980 [84,85,86], and that the mean age of HZO onset has been steadily decreasing, possibly reflecting a weaker life-long immunity acquired from the varicella vaccination compared to the immunity gained from natural infection [87].

The gB and gH/gL glycoprotein complexes are vital in viral attachment, membrane fusion, and effective propagation of VZV through the regulation of cell-to-cell fusion characteristic of VZV pathogenesis. The cell surface receptors heparan sulfate proteoglycans (HSPGs) and mannose-6-phosphate (M6PR) are implicated in VZV entry [97,98]. This virus has the ability to establish permanent latency in the sensory neurons of the dorsal root ganglion and along the entire neuraxis via retrograde or T-cell mediated transport. It can subsequently reactivate in response to a variety of stresses, including fever, trauma, and immunosuppression. The virus then replicates and travels anterograde along the sensory neural pathway, causing neuronal cell damage, demyelination, and a dermatomal vesicular rash. The majority of HZO cases occur secondary to reactivated VZV, termed herpes zoster, as primary VZV infection rarely causes keratitis. Because the nasociliary branch of V1 innerves much of the periocular and superficial ocular structures, VZV can spread to these structures, causing epithelial keratitis, stromal keratitis, and endotheliitis [88,89].

#### 3.2.2. Clinical Manifestations of VZV Endotheliitis

VZV endotheliitis manifests primarily in younger patients [1], with reports of it affecting children as young two years of age [51]. Although typically associated with a reactivation of latent VZV, primary varicella infection can also present with corneal endotheliitis [52,53].

**Figure 5:**
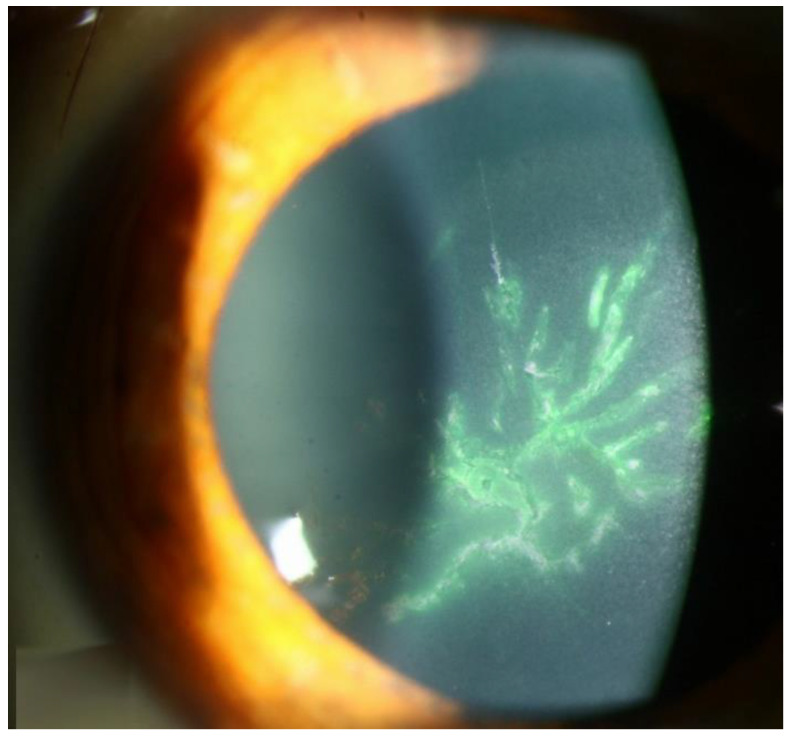
VZV epithelial keratitis pseudodendrites (Courtesy of Welder J and Vislisel J, the University of Iowa, EyeRounds.org (Iowa City, IA, USA); used with the permission of the publisher [99]).

Unique from other viral entities, VZV reactivation is associated with a vesicular eruption on the periocular skin and eyelid along the V1 distribution [52]. Corneal endotheliitis usually presents within 1 month of onset of the painful rash and is preceded by the development of punctate epitheliopathy and pseudodendrite formation, which typically occur within 10 days of skin involvement [54]. Some patients may also develop prolonged epithelial keratitis similar to HSV-associated cases, but in contrast to HSV dendritic keratitis, pseudodendrites in VZV usually lack terminal bulbs and stain poorly with fluorescein and rose bengal (Figure 5) [54].

**Figure 6:**
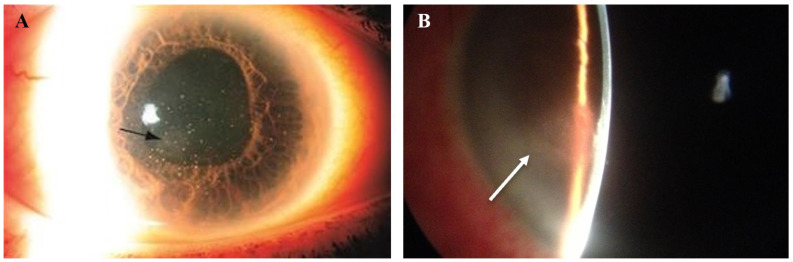
Slit lamp photos of two patients with VZV endotheliitis. (**A**) Diffuse KPs and disciform corneal edema (black arrow) in VZV endotheliitis. (**B**) Disciform corneal edema (white arrow). ((**A**) courtesy of EyeWiki (San Francisco, CA, USA); used with the permission of the publisher [64]).

As with other etiologic variants of viral endotheliitis, the most commonly reported symptoms include photophobia, conjunctival injection, and decreased visual acuity unilaterally [52,55,56,57,58,59]. Manifestations of VZV endotheliitis following the onset of the dermatomal rash include the onset of corneal edema, keratic precipitates, and Descemet folds within 4–7 days (Figure 6) [54]. Similar to CMV and HSV, the corneal edema and KPs are most commonly found in a disciform pattern [52,55,56] but can also manifest diffusely [60], sectorally, or linearly [60]. In comparison with HSV-associated endotheliitis, cases secondary to VZV usually present with a more severe anterior chamber inflammation, with a greater chance of developing a hyphema or hypopyon [6,57,58,59]. Finally, as is the case with CMV and HSV, VZV has also been implicated as a cause of endotheliitis following keratoplasty [60].

#### 3.2.3. Diagnostic Techniques of VZV Endotheliitis

As with any suspected viral endotheliitis, PCR of the aqueous humor from the anterior chamber remains the gold standard [1]. Serologic tests can help confirm the presence or absence of antibodies from a recent infection and are usually performed in conjunction with PCR to confirm diagnosis of VZV. However, serological tests fail to detect an IgM antibody response in about 50% of cases and may require days to weeks after onset of reactivation to show a positive test [61,64]. Finally, since the majority of adults have had primary varicella infection or vaccination, the presence of IgG antibodies against VZV has limited utility in diagnosis [66]. ELISA, enzyme immunoassay, fluorescent antibody to membrane antigen (FAMA), hemagglutination, immune adherence, complement fixation and neutralization to detect VZV-specific IgG, IgA, and IgM responses remain options [61], with FAMA being the gold standard, as it correlates best with immune status against varicella [61,62,63,64,65]. However, limitations to FAMA include its substantial cost, labor-intensive protocol, and the variable expertise in test interpretation.

Given the imperfect diagnostic modalities available, perhaps the most critical and distinguishing factor for accurate diagnosis of a VZV-related endotheliitis is a history of a preceding ipsilateral vesicular skin rash that is characteristic of the virus.

#### 3.2.4. Treatment of VZV Endotheliitis

Initiating treatment as soon as possible is imperative in not only minimizing the risk of ocular complications, but also in improving the overall morbidity associated with VZV infections [56]. As in CMV and HSV, prior studies have supported the use of topical corticosteroids early in the disease process to control the inflammation and prevent irreversible damage to the corneal endothelium, with some suggesting the possibility of a need for a life-long low-dose maintenance therapy [58,67,68,69].

Oral acyclovir 800 mg five times daily for 7–10 days is recommended as systemic antiviral therapy for patients older than 12 years of age [53,56]. Combination therapy consisting of oral acyclovir, a topical steroid such as prednisolone acetate 1%, and a topical cycloplegic until ocular symptoms resolve, followed by slow taper of steroids, may be considered [52,70]. Valacyclovir, a prodrug of acyclovir with 3–5 times greater oral bioavailability as compared to acyclovir, can be dosed at 1 g three times daily, providing the bio-equivalent of the aforementioned dose of acyclovir [52,70]. A role for long-term antiviral therapy has not yet been fully elucidated, although may be considered particularly in chronic or recurrent cases.

In a case report of an immunocompromised patient with concomitant VZV and CMV endotheliitis [71], a regimen involving oral valganciclovir 900 mg twice daily for six weeks followed by 450 mg twice daily for an additional six weeks, along with oral acyclovir 800 mg five times daily for seven days and topical corticosteroids every two hours, was found to completely clear the cornea six weeks later, with complete clearance of KPs and anterior chamber inflammation. During the course of treatment, monthly aqueous taps were performed to closely monitor the titers of VZV DNA and CMV DNA, and the patient was found to be negative for both after four months of systemic therapy. With continued prophylactic acyclovir once a day, the patient remained free of signs and symptoms at follow up visits for 12 months [71]. This highlights the possibility of concomitant infections, especially in the immunocompromised population.

Finally, as in cases of severe corneal decompensation due to CMV and HSV, corneal transplantation may be necessary [14,49].

### 3.3. Herpes Simplex Virus (HSV)

#### 3.3.1. Epidemiology and Pathophysiology of HSV Endotheliitis

Another member of the *Herpesviridae* family, HSV is a double-stranded DNA virus with a recent seroprevalence of 57.7% for HSV type 1 (HSV-1) in the United States [4]. According to a report by Young et al., the yearly incidence of ocular HSV infections is estimated to be 11.8 per 100,000 people in the United States [90]. The majority of ocular HSV infections are caused by HSV-1, with the exception of neonatal ocular infections contracted during descent through an infected birth canal, in which the majority of cases are caused by HSV type 2 (HSV-2) [3].

The glycoproteins gB and gD serve as important immune epitopes for the body to recognize the virus, with initial contact between viral gB and cell surface HSPGs facilitating initial viral attachment and later serving as a membrane fusion protein for the delivery of virus into the cytoplasm [100]. Interactions between gD and its cell receptors on the surface of host membrane result in an activation signal for recruitment of the viral fusion complex. The gD cell receptors Nectin-1, herpesvirus entry mediator (HVEM), and 3-O-sulfated heparan sulfate (3-OS HS) have been well documented in ocular HSV cell lines [101,102,103,104]. HSV is typically confined to dorsal root ganglia, with HSV-1 intermittently reactivating along the trigeminal ganglion. During times of stress, including exposure to ultraviolet light, psychological stress, and hormonal fluctuation, or immune modulation [1,2], the virus then travels anterograde with the branches supplying the trabecular meshwork, iris and ciliary body, causing viral particles to be shed into the anterior chamber and producing recurrent disease. When these viral particles are captured by indigenous antigen-presenting cells (APCs), an immunogenic signal is generated, inducing virus-specific anterior chamber associated immune deviation (ACAID) [42,77,78,79,82]. Finally, data on rat, rabbit, non-human primate, and human tissue presented by Kennedy et al. suggest that the cornea itself may be a reservoir for viral latency, as HSV has been isolated in certain corneal specimens with negative cultures from the corresponding trigeminal ganglion [91]. However, due to the limitations in detecting HSV, further studies are needed to elucidate this theory.

The activation of the endoglycosidase heparanase (HPSE) with the distinctive ability to degrade HS is vital for viral release and subsequent pathogenesis through increased translocation of NF-κB to the nucleus [96,105]. As a trigger for inflammation, HPSE is upregulated by HSV to result in an enhancement of viral spread, inhibition of wound closure, and increased production of pro-inflammatory factors through the destabilization of tissue architecture [103,106,107]. The production of cytokines, chemokines, and inflammatory cells drives the pathogenesis of the infection. In the acute stage, neutrophils are predominant, though natural killer (NK) cells, dendritic cells, and macrophages are also activated. After the initial 7 days, CD4+ T cells become predominant by producing various cytokines such as IFN-g and IL-17, which drive the infiltration and activation of a second wave of neutrophils, which are thought to be even more pathogenic than the first.

#### 3.3.2. Clinical Manifestations of HSV Endotheliitis

**Figure 7:**
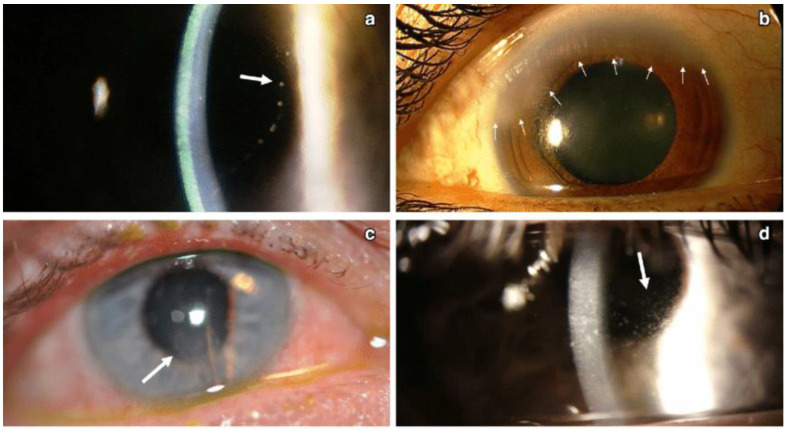
Different patterns of keratic precipitates in viral endotheliitis. (**a**) Linear: fine KP (white arrow) in a linear pattern overlying corneal edema. (**b**) Sectoral: HSV endotheliitis with sectoral corneal edema (white arrows). (**c**) Disciform: a disciform pattern of KP (white arrow) with overlying corneal edema. (**d**) Diffuse: endotheliitis presenting with diffuse KP (white arrow) (Courtesy of Dr. Majid Moshirfar; used with the permission of the publisher [1]).

**Figure 8:**
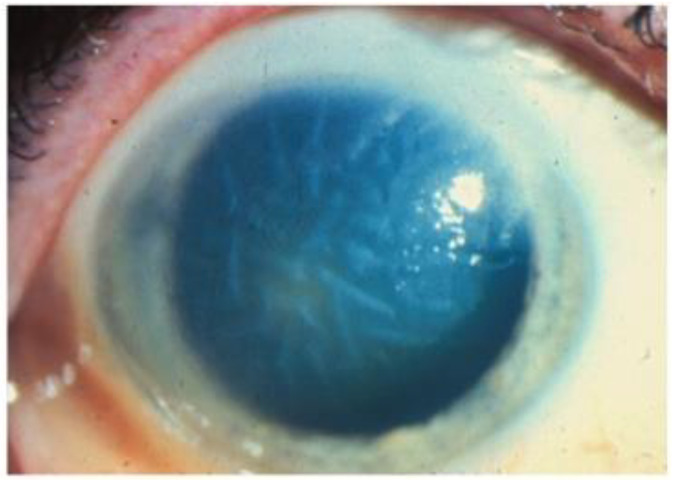
Stromal edema due to HSV endotheliitis, with endothelial dysfunction in the absence of neovascularization and stromal inflammation (courtesy of EyeWiki; used with the permission of the publisher [21]).

Unlike CMV, HSV endotheliitis occurs more commonly in individuals who are immunocompromised and in those with prior ocular infection [1]. Generally, HSV endotheliitis is caused by HSV-1, but HSV-2 may also be implicated [16]. Roughly one-third of HSV endotheliitis cases are bilateral, and like in CMV, a disciform pattern of KPs is most commonly found, although other configurations are possible (Figure 7) [19]. Similarly to CMV and VZV, overlying corneal edema may develop, severely limiting visual acuity (Figure 8) [19].

Because HSV endotheliitis following endothelial keratoplasty has been reported, it is crucial to distinguish between primary graft failure and viral corneal endotheliitis [17]. Matar et al. report a case of a patient with Fuchs’ endothelial corneal dystrophy (FECD) along with ocular hypertension and cystoid macular edema who underwent three Descemet membrane endothelial keratoplasties (DMEKs) due to presumed recurrent primary graft failures. At the third DMEK, however, HSV-1 was detected in the aqueous humor and antiviral therapy was initiated; at the 6-month follow-up, the cornea remained clear, visual acuity remained stable, IOP had normalized, and macular edema had regressed completely [75].

#### 3.3.3. Diagnostic Techniques of HSV Endotheliitis

As with CMV and VZV, PCR and ELISA analyses of the aqueous humor can be performed for the diagnosis of HSV-related endotheliitis, bearing in mind that yield is limited in cases with mild anterior chamber reactions and in late stages of infection and that a negative result does not exclude a viral etiology [18,25,26,27]. The combination of the two modalities may provide a higher yield [28].

Real-time PCR with the use of LightCycler, a combination of spectrofluorophotometer and thermal cycler, quantifies the amount of DNA accurately in the range of exponential amplification [22,23]. Furthermore, it can differentiate between various HSV types, with a report by Corey et al. suggesting that both HSV-1 and HSV-2 can be detected as long as the quantitative difference is within 1000-fold [23,24]. If the difference is between 1000 and 10,000-fold, then the more prevalent strain will be detected [24].

Confocal microscopy may show pseudoguttae with inflammatory cell infiltration, enlargement of intercellular gaps, and a decrease in endothelial cell density, but these changes are not specific to HSV [15,29]. Unlike CMV, confocal microscopy for HSV does not reveal any pathognomonic findings specific to HSV, limiting its utility [9].

#### 3.3.4. Treatment of HSV Endotheliitis

Given the inflammatory nature of the condition, topical steroids, in addition to antivirals, are recommended in the treatment of herpetic endotheliitis. A number of randomized, double-masked trials compared the combination of topical betamethasone (0.1% or 0.01%) and topical acyclovir 3% ointment with acyclovir ointment alone [30], all of which concluded that the combination of a topical steroid with a topical antiviral produced a faster response and significantly fewer treatment failures than treatment with topical acyclovir alone [30]. One study by Porter et al. compared the use of topical acyclovir 3% five times daily vs. oral acyclovir 400 mg five times daily in the treatment of herpetic endotheliitis and found no significant difference in the average time to healing or the incidence of recurrence over a three-year post-treatment period between the two groups [30]; however, oral acyclovir showed greater improvement in visual acuity [30].

While medical therapy has been shown to be efficacious in many patients with herpetic endotheliitis, severe cases with persistent corneal edema or corneal scarring may require surgical intervention [31]. In cases with concurrent anterior stromal scarring, patients may benefit from full-thickness penetrating keratoplasty (PK). DMEK has been reported in cases of isolated endotheliitis [13,32]. One retrospective case series comparing the outcomes of 17 eyes with herpetic endotheliitis treated with DMEK with 72 eyes with FECD or pseudophakic bullous keratopathy (PBK) also treated with DMEK showed that DMEK surgery significantly improved best corrected visual acuity (BCVA) in patients herpetic endotheliitis, but the rate of postoperative complications, including graft failure, corneal ulcers, and cystoid macular edema, was significantly higher in the herpetic endotheliitis group as compared to the FECD and PBK groups [73]. As such, a course of systemic valacyclovir should be considered after surgical intervention to reduce the risk of HSV-1 recurrence and graft failure [31].

## 4. Discussion

**Figure 9:**
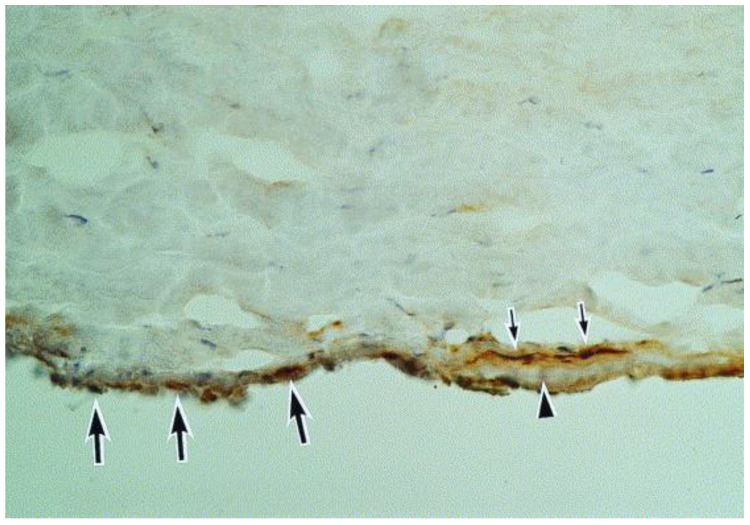
A section of the Descemet membrane (arrowhead) and the tip of the trabeculum. Immunoreactivity for herpes simplex virus can be seen in the trabeculum (large arrows) and in the keratocytes (small arrows) adjacent to the Descemet membrane (hematoxylin and eosin, ×40) (Courtesy of Amano et al.; Used with the permission of the publisher [21]).

We have summarized here the clinical presentation, pathogenesis, and management of HCE (Table 1). While it is challenging to cover all relevant topics, it is important to consider potential ocular complications that may result from this condition. HCE may be a direct result of viral activity, an inflammatory response to viral presence, or a combination of both. Notably, because HCE can often present with elevated intraocular pressure (IOP), presumably due to inflammation of the trabecular meshwork (Figure 9) [20,21], prompt recognition and treatment may prevent glaucomatous optic neuropathy, a cause of permanent visual morbidity. In fact, the aforementioned herpesviruses have been associated with Posner–Schlossman syndrome and Fuchs’ heterochromic iridocyclitis [10,33,34].

As we have described above, specular microscopy may be considered in some HCE cases, but unfortunately, this imaging modality is not widely available and interpretation can be difficult. Polymerase chain reaction (PCR) testing of the aqueous humor can be performed, although the diagnostic yield may be limited in cases with mild anterior chamber reactions and in late stages of infection. The Goldmann–Witmer coefficient (GWC), by comparing intraocular and serum antibody levels, can be utilized as an adjunctive tool to PCR when the diagnosis is open to question, but it is of limited utility amongst immunocompromised patients due to low total antibody production [37,39]. Finally, metagenomic next generation sequencing may be a promising strategy, but still requires further validation [72].

In particular, differentiating between HCE and corneal graft rejection is of substantial clinical importance, as the former is treated with antivirals and steroids, while the latter is treated with steroids/immunosuppression alone; a lack of appropriate antiviral therapy in cases of HCE can lead to graft failure. Thus, if a patient has an early graft failure or a history of multiple graft failures [74,75], an anterior chamber paracentesis for PCR testing should be considered. The importance of this distinction is illustrated by Anshu et al., who reported a series of four patients with undiagnosed CMV endotheliitis after endothelial keratoplasty, who did not receive antiviral therapy until further corneal compromise and/or retinitis developed [76]. Morishige et al. likewise recognized the importance of this distinction and noted that changes in IOP and corneal manifestations may provide a basis for differentiation of viral endotheliitis from allograft rejection in individuals with anterior chamber inflammation after keratoplasty and may thus allow for initiation of appropriate treatment before viral DNA is identified [60].

## 5. Conclusions

HCE is a sight-threatening condition with potentially permanent consequences. Given the corneal endothelium’s limited ability to regenerate after insults, the management of viral endotheliitis can be a particularly critical task. With prompt recognition and proper treatment, the visual and ocular morbidity, as well as the need for surgical intervention, can potentially be reduced. In this review article, we have discussed the present literature with the aim of aiding clinicians in managing patients affected by this challenging condition.

## Figures and Tables

**Table 1:** Summary of main takeaway points about epidemiology, clinical manifestation, diagnosis, and treatment of CMV, VZV, and HSV as discussed in this review paper.

	Epidemiology	Clinical Manifestation	Diagnosis	Treatment
Cytomegalovirus (CMV)	Seroprevalence varies between 45 and 100% Most commonly seen in middle-aged and older men Asymptomatic primary infection, lifelong latency in bone marrow and stem cells Typically affects healthy patients Posterior segment infection more common in immunocompromised individuals	Small to medium, pigmented, non-granulomatous keratic precipitates Linear, sectoral, disciform, or diffuse pattern of keratic precipitates	Primarily a clinical diagnosis PCR from anterior chamber tap may be considered Presence of “owl’s eye” inclusion bodies is pathognomonic Confocal microscopy may be considered (may show owl’s eye configuration) Serologic tests may be considered	Topical corticosteroid in combination with a topical and/or systemic antiviral Topical antiviral regimen: 0.15% ganciclovir gel 5 times daily with subsequent taper Systemic antiviral regimen: oral valganciclovir 900 mg BID with subsequent taper IOP-lowering medications indicated in cases of ocular hypertension Corneal transplantation in cases of irreversible corneal decompensation
Varicella Zoster Virus (VZV)	Seroprevalence is 95–99% Ocular involvement in 10–20% of VZV infection due to V1 nerve involvement Asymptomatic primary infection, lifelong latency in sensory nerve ganglia	Vesicular eruption on the periocular skin and eyelid along the V1 distribution Corneal endotheliitis may present within 1 month of onset of the painful rash Linear, sectoral, disciform, or diffuse pattern of keratic precipitates	Primarily a clinical diagnosis PCR from anterior chamber tap may be considered Serologic tests may be considered	Topical corticosteroid in combination with a systemic antiviral Systemic antiviral regimen: valacyclovir 1000 mg PO TID with subsequent taper IOP-lowering medications indicated in cases of ocular hypertension Corneal transplantation in cases of irreversible corneal decompensation
Herpes Simplex Virus (HSV)	Seroprevalence is 58% Majority of ocular HSV is caused by HSV-1 except neonatal ocular infection which is typically caused by HSV-2	Prior history of dendritic keratitis is suggestive bilaterally Disciform pattern of keratic precipitates is most common (may also be linear, sectoral, or diffuse)	Primarily a clinical diagnosis PCR from anterior chamber tap may be considered Serologic tests may be considered	Topical corticosteroid in combination with a systemic antiviral Systemic antiviral regimen: valacyclovir 500 mg PO TID with subsequent taper IOP-lowering medications indicated in cases of ocular hypertension Corneal transplantation in cases of irreversible corneal decompensation

## Data Availability

No new data were created or analyzed in this study. Data sharing is not applicable to this article.
